# Correction: Neural adaptation and fractional dynamics as a window to underlying neural excitability

**DOI:** 10.1371/journal.pcbi.1011220

**Published:** 2023-06-12

**Authors:** Brian Nils Lundstrom, Thomas J. Richner

In ‘Understanding multiple timescale adaptation as fractional differentiation’ subsection of the Results, there is an error in the equation on page 6. Please view the complete, correct equation here[1]:

$$D^{\alpha }f(x)=\frac{1}{\Gamma (1-\alpha )}\frac{d}{dx}\int_{0}^{x}{\left(x-t\right)}^{-\alpha }f(t)dt$$
