# Supplementary material for: Neural adaptation and fractional dynamics as a window to underlying neural excitability
Source: PLoS Comput Biol. 2023 Feb 21;19(2):e1010527. doi: 10.1371/journal.pcbi.1010527 (PMC9983885; doi:10.1371/journal.pcbi.1010527)
Supplement: S1 Text — (DOCX) [file pcbi.1010527.s001.docx]

**Supplemental Materials**

The following models assume that firing rates are greater than zero, inputs are slowly varying, and outputs are a linear function of inputs over some regime [1–4].

**Spike Frequency Adaptation**

A neuron model for action potential firing rates as a function of time can be written:

$$\begin{aligned} y=\gamma x - ca \\ \tau\frac{da}{dt}&=-a+Fy \#\#\#\# \end{aligned}$$

(1)

where *y* is the firing rate output, *x* is the mean current input, *a* is the adaptation variable, *c* and γ are positive gain constants, *F* governs the amount of adaptation, and *τ* is the effective adaptation time constant [1,2], which is shorter than the time constant of the underlying mechanism [5].

The firing rate output is assumed to be non-negative. Using the Laplace transform we find the transfer function *H(s)*:

$$a=1/c \left( \gamma x-y \right)$$

$$\tau sa=-a+Fy$$

$$\tau s/c\left( \gamma x-y \right)=-1/c\left( \gamma x-y \right)+Fy$$

$$\tau sy + y +\mathrm{cFy}= \tau s\gamma x + \gamma x$$

$$y\left( \tau s+1+cF \right)=\gamma x\left( \tau s+1 \right)$$

$$H\left( s \right)=\gamma\frac{\tau s+1}{\tau s+1+cF}$$

(2)

**Synaptic Depression**

A model for synaptic responses that include synaptic depression can be written as:

$$y=\gamma xa$$

$$\tau\frac{da}{dt}=1-a - xFa$$

(3)

where *y* is the synaptic membrane, *x* is the firing rate input, *a* is the adaptation variable related to the readily releasable pool of synaptic vesicles, *c* and γ are gain constants, *F* governs the amount of adaptation, and *τ* is the effective adaptation time constant. Due to the nonlinearity of the term *xa*, these equations are linearized around a fixed point (*a_0_ ,* *x_0_*), as has been discussed previously [3]. Here, we let *a* and *x* now represent small differences (ie δa, δx) from steady-state values *a_0_* and *x_0_* such that linearized equations $L\left( x,a \right)=f\left( x_{0},a_{0} \right)+f_{x}\left( x_{0},a_{0} \right)\delta x+f_{a}\left( x_{0},a_{0} \right)\delta a$ are:

$$y=\gamma x_{0}a+\gamma xa_{0}$$

$$\tau\frac{\mathrm{da}}{\mathrm{dt}}=-Fa_{0}x - a - x_{0}\mathrm{Fa}$$

(4)

Note that the *f(x_0_*_,_ *a_0_,*) term does not appear since now *a=δa*, and *x=δx*. Using the initial equation for *a*, we note that at steady-state: $a_{0}\left( 1+x_{0}F \right)=1$. Using the Laplace transform we find the transfer function *H(s)*:

$$a=\left( y-\gamma xa_{0} \right)/\left( \gamma x_{0} \right)$$

$$\tau s\left( y-\gamma xa_{0} \right)=-\gamma x_{0}Fa_{0}x-\left( y-\gamma xa_{0} \right)-x_{0}F\left( y-\gamma xa_{0} \right)$$

$$y\left( \tau s+1+x_{0}F \right)=\gamma a_{0}x\left( \tau s-x_{0}F+1+x_{0}F \right)$$

$$H\left( s \right)=\gamma a_{0}\frac{\tau s+1}{\tau s+1+x_{0}F} =\frac{\gamma}{1+x_{0}F}\left( \frac{\tau s+1}{\tau s+1+x_{0}F} \right)$$

(5)

**Power and Phase of the Transfer Function**

The general form of the transfer functions for spike frequency adaptation and synaptic depression, where *A>*1, is:

$$H\left( s \right)=g\frac{s+1}{s+A}$$

(6)

The power for this transfer function (with *s=iω*) is:

$$H\left( i\omega\right)=g\frac{\tau^{2}\omega^{2}+A+i\left( \tau\omega A-\tau\omega\right)}{\tau^{2}\omega^{2}+A^{2}}$$

$$\left| H\left( i\omega\right) \right|^{2}=g^{2}\frac{\left( \tau^{2}\omega^{2}+A \right)^{2}+\left( \tau\omega A-\tau\omega\right)^{2}}{\left( \tau^{2}\omega^{2}+A^{2} \right)^{2}}$$

$$\left| H\left( i\omega\right) \right|^{2}=g^{2}\frac{\left( \tau^{2}\omega^{2}+A^{2} \right)\left( \tau^{2}\omega^{2}+1 \right)\left( \{\tau\left( \tau\right\}^{2}\omega^{2}+1 \right)}{\left( \tau^{2}\omega^{2}+A^{2} \right)^{2}}=g^{2}\frac{\tau^{2}\omega^{2}+1}{\tau^{2}\omega^{2}+A^{2}}$$

(7)

The phase for this transfer function is:

$$\angle H\left( i\omega\right)=\arctan\left( \frac{\tau\omega A-\tau\omega}{\tau^{2}\omega^{2}+A} \right)$$

(8)

For small angles, this can be simplified to:

$$\angle H\left( i\omega\right)\approx\frac{\tau\omega\left( A-1 \right)}{\tau^{2}\omega^{2}+A}$$

(9)

**Properties of Fractional Derivatives**

Fractional derivatives are defined by a power law gain response (increasing amplitude with decreasing frequency) and frequency independent phase shift of *απ/2*, where *α* is the order of the derivative. The derivation uses Euler’s identity *exp*(*iπ/2) = i sin(π/2) = i* as below:

$$sX\left( \omega\right)=\left( i\omega\right)^{\alpha}X\left( \omega\right)$$

$$=\left( \omega\right)^{\alpha}e\mathrm{xp}\left( i\frac{\alpha\pi}{2} \right)C_{\omega}e\mathrm{xp}\left( i\theta_{\omega} \right)$$

$$=\left( \omega\right)^{\alpha}C_{\omega}e\mathrm{xp}\left( i\theta_{\omega}+i\frac{\alpha\pi}{2} \right)$$

(10)

**Approximating Multiple Adaptation Mechanisms with High-pass Filters**

For multiple adaptation process in series, the adaptive elements are multiplied in the frequency domain:

$$H\left( s \right)=\prod_{n} g_{n}\frac{{}_{n}s+1}{{}_{n}s+A_{i}}$$

(11)

Where *A = 1+cF* for mechanisms of the same form as SFA. Alternately, one may consider a series of adaptive mechanisms, such as adaptive currents *a*, that affect spike generation:

$$y=\gamma x - \sum\mathrm{ca}$$

$$H\left( s \right)=\frac{\gamma}{1+\sum\frac{\mathrm{cF}}{\tau s+1}}$$

(12)

In this case, the adaptive elements are not completely in series [2]. However, in the end the results between the two forms are similar. The only difference in the resulting rational polynomial transfer functions is in the zeroth order term in the denominator. For the first (multiplicative) case the final term is $\prod_{n} A_{n}$ whereas for the second (additive) case the term is $\sum_{n} A_{n}-\left( n-1 \right)$. For typical values of *A*, this does not significantly affect results.

**Approximating Fractional Differentiation with Multiple High-pass Filters**

Adaptation emphasizes stimulus change and can be approximated by high-pass filters. For example, for three timescales of adaptation the transfer function is:

$$H\left( s \right)=g_{1}\left( \frac{\tau_{1}s+1}{\tau_{1}s+A_{1}} \right)g_{2}\left( \frac{\tau_{2}s+1}{\tau_{2}s+A_{2}} \right)g_{3}\left( \frac{\tau_{3}s+1}{\tau_{3}s+A_{3}} \right)$$

(13)

For simplicity we assume that *τ_3_ = 10τ_2_ = 100τ_1_* and *A_1_ = A_2_ = A_3_* and *g = g_1_ g_2_ g_3_.*

$$H\left( s \right)=g\left( \frac{\tau s+1}{\tau s+A} \right)\left( \frac{10\tau s+1}{10\tau s+A} \right)\left( \frac{100\tau s+1}{100\tau s+A} \right)$$

(14)

For three timescales as above, the phase is:

$$\angle H\left( i\omega\right)\approx\frac{\tau\omega\left( A-1 \right)}{\tau^{2}\omega^{2}+A}+\frac{10\tau\omega\left( A-1 \right)}{100\tau^{2}\omega^{2}+A}+\frac{100\tau\omega\left( A-1 \right)}{10,000\tau^{2}\omega^{2}+A}$$

(15)

High-pass filters that approximate adaptation give rise to phase advances [2]. Multiple high-pass filters can approximate multiple timescale adaptation. And to the extent that they approximate fractional dynamics, they have frequency-independent phase. These filters are minimum phase systems, where specifying the phase determines the magnitude to within a constant factor [6,7]. A rational linear time-invariant system is minimum-phase if it and its inverse are causal and stable. Practically, this means that minimizing the following function yields an approximation between fractional dynamics and the rational filters of adaptation such that *A_i_* can be determined for a given set of *τ_i_* :

$$\mathrm{argmi}n_{A_{i}}\left( \angle s^{\alpha}-\angle\prod_{i} g_{i}\frac{\left( \tau_{i}s+1 \right)}{\left( \tau_{i}s+A_{i} \right)} \right)$$

$$min\_\{A\_i\}├(\{Ⅎrangles\}^\alpha-\angle\prod\_\{i\}\{g\_i⍁\{├(\tau\_is+1┤)\}\{├(\tau\_is+A\_i┤)\}\}┤)$$

$$=arg\mathrm{mi}n_{A_{i}}\left( \alpha\pi/2-\prod_{i} \frac{\left( \tau_{i}\omega\left( A_{i}-1 \right) \right)}{\left( \tau_{i}^{2}\omega^{2}+A_{i} \right)} \right)$$

(16)

**References**

1. Benda J, Herz AV. A universal model for spike-frequency adaptation. Neural Comput. 2003;15: 2523–2564.

2. Lundstrom BN. Modeling multiple time scale firing rate adaptation in a neural network of local field potentials. Journal of Computational Neuroscience. 2015;38: 189–202. doi:10.1007/s10827-014-0536-2

3. Tripp BP, Eliasmith C. Population models of temporal differentiation. Neural Comput. 2010;22: 621–659. doi:10.1162/neco.2009.02-09-970

4. Varela JA, Sen K, Gibson J, Fost J, Abbott LF, Nelson SB. A quantitative description of short-term plasticity at excitatory synapses in layer 2/3 of rat primary visual cortex. J Neurosci. 1997;17: 7926–7940.

5. Liu YH, Wang XJ. Spike-frequency adaptation of a generalized leaky integrate-and-fire model neuron. J Comput Neurosci. 2001;10: 25–45.

6. Oppenheim AV, Schafer RW, Buck JR. Discrete-time signal processing. 2nd ed. Upper Saddle River, N.J.: Prentice Hall; 1999.

7. Ulrych TJ, Lasserre M. Minimum-Phase. Canadian Journal of Exploration Geophysics. 1966;2: 22–32.
